# Inhibition of gold nanoparticles (AuNPs) on pathogenic biofilm formation and invasion to host cells

**DOI:** 10.1038/srep26667

**Published:** 2016-05-25

**Authors:** Qilin Yu, Jianrong Li, Yueqi Zhang, Yufan Wang, Lu Liu, Mingchun Li

**Affiliations:** 1 Key Laboratory of Molecular Microbiology and Technology, Ministry of Education, Department of Microbiology, Nankai University, Tianjin, PR China; 2 Clinical Laboratory, Tianjin Third Central Hospital, Tianjin, PR China; 3 Tianjin Key Laboratory of Environmental Remediation and Pollution Control, College of Environmental Science and Engineering, Nankai University, Tianjin, PR China

## Abstract

Owing to the growing infectious diseases caused by eukaryotic and prokaryotic pathogens, it is urgent to develop novel antimicrobial agents against clinical pathogenic infections. Biofilm formation and invasion into the host cells are vital processes during pathogenic colonization and infection. In this study, we tested the inhibitory effect of Au nanoparticles (AuNPs) on pathogenic growth, biofilm formation and invasion. Interestingly, although the synthesized AuNPs had no significant toxicity to the tested pathogens, *Candida albicans* and *Pseudomonas aeruginosa*, the nanoparticles strongly inhibited pathogenic biofilm formation and invasion to dental pulp stem cells (DPSCs). Further investigations revealed that AuNPs abundantly bound to the pathogen cells, which likely contributed to their inhibitory effect on biofilm formation and invasion. Moreover, treatment of AuNPs led to activation of immune response-related genes in DPSCs, which may enhance the activity of host immune system against the pathogens. Zeta potential analysis and polyethylene glycol (PEG)/polyethyleneimine (PEI) coating tests further showed that the interaction between pathogen cells and AuNPs is associated with electrostatic attractions. Our findings shed novel light on the application of nanomaterials in fighting against clinical pathogens, and imply that the traditional growth inhibition test is not the only way to evaluate the drug effect during the screening of antimicrobial agents.

Owing to the growing infectious diseases caused by various eukaryotic and prokaryotic pathogens, development of antimicrobial agents is one of the most significant aspects for decreasing pathogenic dangers^1^,^2^,^3^. As time passes, nanotechnology has advanced in almost all fronts, especially in industry, agriculture, energy, environmental protection and medicine^4^,^5^,^6^. Many nanomaterials (NMs), such as silver nanoparticles (AgNPs)^7^,^8^, carbon nanotubes (CNT)^9^, graphene oxides (GO)^10^,^11^, fullerenes^12^, titanium dioxide (TiO_2_) NMs^13^, iron NPs^14^, zinc oxide (ZnO) NPs^15^, gold NPs (AuNPs)^16^, have been demonstrated to have antimicrobial activity. These NMs inhibit or kill microbes by releasing metal ions, disrupting cell membrane or cell wall, producing ROS, damaging DNA or mitochondria, etc^7^,^17^. However, although most of them have efficient activity against prokaryotic pathogens, their effects and mechanisms against eukaryotic pathogens are poorly understood.

Eukaryotic pathogens, mainly including pathogenic fungi and protists, have worldwide distribution, high morbidity and high mortality in the clinic. *C. albicans*, one of the most important eukaryotic pathogens, ranks as the fourth-greatest cause of bloodstream infections^18^,^19^. Moreover, this fungus, together with many other opportunistic pathogens, commonly colonizes in human orals. When the host immune defense system is weakened, it may cause various types of oral candidiasis, mainly including pulpitis, thrush and angular cheilitis^20^,^21^,^22^. With the prevalence of risk factors, such as neutropenia, antibiotic treatment, organ transplantation and long-time intensive care unit (ICU) stay, candidiasis-related diseases have been of great concern^23^,^24^. Most of antifungal drugs target the synthesis and function of ergosterol required for *C. albicans*. However, their efficacy is compromised by the emergence of drug resistant strains and the strong ability of this fungus in biofilm formation^25^,^26^. Therefore, it is urgent to develop novel and efficient agents and strategies against this eukaryotic pathogen, especially to explore those which have impact on its virulence factors.

Both eukaryotic and prokaryotic pathogens possess a wide range of virulence factors for their infections, including biofilm formation, invasion to host cells, morphological transition, and stress response^27^,^28^. Among those factors, detailed investigations on biofilm formation and invasion are particularly meaningful, not only due to the current insufficiency in uncovering the mechanisms regulating those processes, but also for their significance in pathogenicity. On the surfaces of specific substrates, such as mucosa, teeth and artificial implanted devices, the pathogens form biofilms which confer them strong resistance to various environmental stresses^29^,^30^. Biofilm formation begins with the adherence of pathogen cells to a substrate, a key process requiring the action of abundant adhesins. During biofilm development, the adhered cells proliferate on the surface and accumulate extracellular matrix. This complex structure is strongly resistant to extracellular stress and has high stability of colonization^31^,^32^. On the other hand, for the invasion process, which is the key process for life-threatening systemic infections, the colonized pathogenic cells switch to invasive status when the host immune system is compromised. Then invasion process originates from sensing the signals of epithelial or other touched host cells, followed by penetration, endocytosis and invasion. Owing to the significance of biofilm formation and invasion to the pathogenicity, it may be efficient to block these processes by novel antimicrobial agents.

In this study, as demonstrated in the schematic of this study (Fig. S1), we synthesized two kinds of AuNPs with similar characteristics, and investigated their effect on growth, biofilm formation and invasion of common pathogens, especially of *C. albicans*. To our surprise, the as-synthesized AuNPs had no obvious impact on pathogenic growth, but strongly inhibited pathogenic biofilm formation and invasion, and activated host immune response. Moreover, these inhibitory effects are associated with the electrostatic attractions between AuNPs and their targeted cells. This study for the first time uncovered the inhibitory effect of AuNPs against pathogenic biofilm formation and invasion, and explored a novel mechanism by which NMs attenuate the virulence of pathogens.

## Results

### Characteristics of the synthesized AuNPs

**Figure 1:**
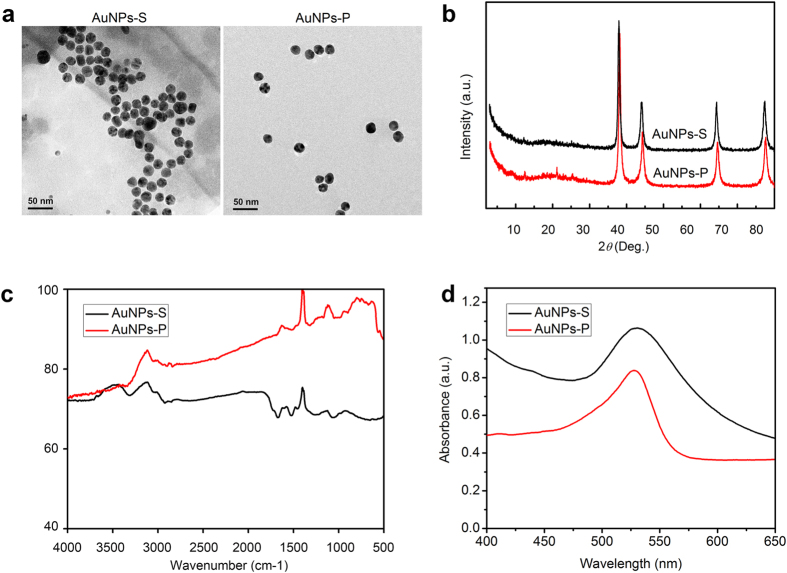
Characterization of the as-synthesized AuNPs. (**a**) TEM observation of AuNPs-S (synthesized using apple extract by Sharma’s method) and AuNPs-P (synthesized using trisodium citrate by Polte’s method). (**b**) X-ray diffraction patterns. (**c**) FI-TR analysis. (**d**) UV-vis spectra.

By reducing HAuCl_4_ to Au^0^ with apple extract or trisodium citrate, we prepared water-soluble AuNPs, named AuNPs-S and AuNPs-P, respectively. TEM showed that most of the prepared AuNPs were spherical and monodisperse with an average size of 10–20 nm (Fig. 1a). X-ray differentiation (XRD) analysis confirmed that the obtained AuNPs were pure Au^0^ monocrystal structures (JCPDS file no: 04-0784) (Fig. 1b). The possible functional groups of the as-synthesized AuNPs were analyzed by Fourier Transform Infrared Spectrometry (FTIR). The major stretching frequencies of AuNPs-S are 3311.88, 2922.72, 2857.21, 1671.57, 1601.62, 1522.77, 1256.50 and 1059.93 cm^−1^ (Fig. 1c). This indicated the appearance of carbonyl groups, which might be originated from organic acids. In the water solution, the AuNPs had λ_max_ of absorption at 535–555 nm, further confirming the formation of AuNPs (Fig. 1d).

### AuNPs have no obvious inhibitory effect on growth and hyphal development of pathogens

**Figure 2:**
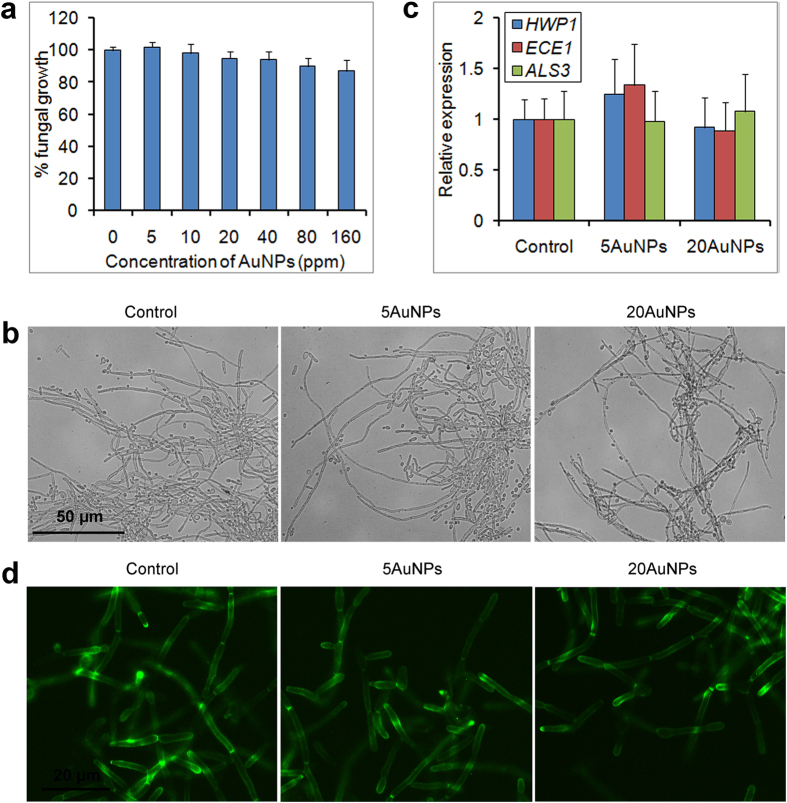
Normal growth and hyphal development of *C. albicans* treated by AuNPs. (**a**) The *C. albicans* cells were treated by AuNPs-S with different concentrations for 24 h, and cellular metabolic activity was determined by MTT assays. (**b**) Normal hyphal development of the fungal cells after 24 h of AuNP treatment. (**c**) Expression levels of hyphal-specific genes after 24 h of treatment. (**d**) Regular cell wall-localization of the hyphal-specific protein Hwp1 observed by fluorescence microscopy. The error bars indicate one standard deviations (n = 3).

Previous study revealed that AuNPs have a potential antifungal activity, which is associated with inhibition to H^+^-ATPase^33^. Herein, we also determined the effect of as-synthesized AuNPs to one of the most important fungal pathogens, *C. albicans*. Both the standard *C. albicans* strains, SC5314 and BWP17, and several clinical isolated strains were used for growth inhibition test. Surprisingly, the AuNPs had no obvious impact on growth of all those strains, even when its concentration reached up to 160 ppm (Fig. 2a), and the IC_50_ of AuNPs to the growth of those strains were >160 ppm.

We also tested the inhibitory effect of AuNPs to *C. albicans* hyphal development, which is essential for pathogenicity of this pathogen^34^,^35^. After 24 h of hyphal induction, AuNPs once again did not affect hyphal development (Fig. 2b). RT-PCR further revealed that the tested hypha-specific genes, *HWP1, ECE1* and *ALS3*, had normal expression levels after AuNP treatment (Fig. 2c). Moreover, when tagged with GFP, the hypha-development protein Hwp1 had normal cell wall- and hyphal tip-localization after this treatment (Fig. 2d). Thus, AuNPs treatment had no effect on *C. albicans* hyphal development, and did not affect the expression and transport of hypha-specific factors.

### AuNPs strongly inhibit *C. albicans* biofilm formation

**Figure 3:**
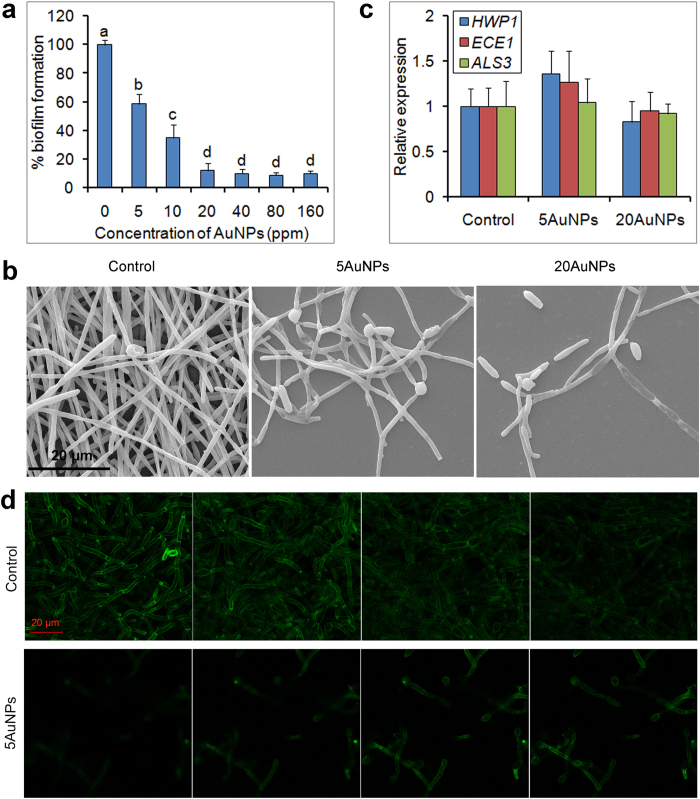
Impaired biofilm formation of *C. albicans* caused by AuNPs treatment. (**a**) *C. albicans* biofilms were formed in polystyrene microtiter plates containing different concentrations of AuNPs, and biofilm activity was measured by XTT assays. (**b**) AuNPs-treated *C. albicans* biofilms observed by scanning electron microscopy. (**c**) Normal expression levels of adhesin genes in the biofilms after 24 h of treatment. (**d**) Defective localization of the adhesin Hwp1 adhered to the microplate bottom surface, which was observed by confocal microscopy. The error bars indicate one standard deviations (n = 3). Identical letters indicate no statistical differences among treatments (P < 0.05).

Pathogenic biofilm formation is important for steady colonization in the host tissues and resistance to environmental stresses, such as antifungal agents and oxidative stress^36^,^37^. Although AuNPs did not affect both growth and hyphal development of *C. albicans*, we further investigated their effect on biofilm formation of this pathogen. Excitingly, 3-(4,5-dimethyl-2-thiazolyl)- 2,5-diphenyl-2-H-tetrazolium bromide (MTT) reduction assays demonstrated that AuNPs significantly inhibited biofilm formation. 5 ppm of AuNPs were sufficient to cause a remarkable decrease in the metabolic activity of biofilms, and ≥20 ppm of AuNPs resulted in >80% decrease (Fig. 3a). The IC_50_ for biofilm formation of the tested strains were 5–10 ppm (Table 1). TEM observation further revealed that biofilm density was significantly reduced under treatment of AuNPs (Fig. 3b), confirming that AuNPs have an impact on biofilm formation in *C. albicans*. Moreover, the two kinds of AuNPs (AuNPs-S and AuNPs-P) had similar inhibitory effect on biofilm formation (Table 1), indicating that the synthesis methods had no impact on this effect. In the following experiments, AuNPs-S were used for further investigations.

We then explored the inhibition mechanisms of AuNPs against *C. albicans* biofilm formation. During biofilm formation, expression of abundant hypha-specific genes, such as the genes tested above, are up-regulated. Their products, namely adhesins, play an important role in adhesion^38^. However, treatment of AuNPs also had no obvious effect on the expression of those hypha-specific genes during biofilm formation (Fig. 3c), indicating that the inhibitory effect of AuNPs on biofilm formation is not attributed to the abnormal expression of biofilm-associated genes. In addition, apple extract alone had no obvious effect on both hyphal development and biofilm formation (data not shown), indicating that the inhibition of AuNPs to biofilm formation is not associated with the appearance of surface organics originated from the apple extract.

Adhesion of the fungal cells to the substrate surfaces is the first key process during biofilm formation, and this process requires abundant adhesins, such as Hwp1 and ALS proteins^39^,^40^. By layer scanning using confocal microscopy, we detected the distribution of the representative adhesin, Hwp1, in the formed biofilms. In the control wells, the adhesin closely adhered to the bottom of the substrate surface, with strong cell wall-localized Hwp1-GFP fluorescence detected in the bottom layer. When examination was performed far from the bottom, GFP fluorescence was also detected in the following layers, indicating that thick biofilms were formed in the control wells (Fig. 3d, the top). In contrast, in the AuNPs-treated wells, the adhesin was not closely attached to the bottom surface, and only faint fluorescence was detected in the bottom layer. Unlike the control wells, strong fluorescence was detected in the third and forth layers rather than the first and the second layers in the AuNPs-treated wells (Fig. 3d, the bottom). This suggested that the nanoparticles attenuated the interaction between the adhesin and the bottom surface, and then resulted in increased planktonic growth and decreased adhesion of the fungal cells to the substrate.

### AuNPs also strongly inhibit biofilm formation of pathogenic bacteria

Besides *C. albicans* and many other eukaryotic pathogens, prokaryotic pathogens, such as *P. aeruginosa*, may also form biofilms for colonization^41^,^42^. Similar to *C. albicans, P. aeruginosa* is widely distributed in the human body, causing nosocomial infections in immunecompromised patients and especially in individuals with severe burns^43^,^44^. This pathogen is a model bacterium for biofilm studies, also displaying high resistance to antimicrobial treatments and to host immune defences^45^,^46^. Here, we further tested the effect of as-synthesized AuNPs on biofilm formation of *P. aeruginosa*. AuNPs showed significant inhibitory effect only when its concentration reached to 10 ppm (IC_50_ = 68.56–75.01 ppm) (Fig. S2a, Table 1). However, 5 ppm of AuNPs strongly inhibited biofilm formation (IC_50_ = 6.851–6.937 ppm) (Fig. S2b, Table 1). Hence, AuNPs also strongly attenuated biofilm formation of the pathogenic bacterium.

### AuNPs inhibit invasion of dental pulp stem cells (DPSCs)

Invasion into host cells is another important process for *C. albicans* pathogenicity, and is required for its systemic infections^47^,^48^. Therefore, inhibition to invasion may be a useful approach for antifungal treatment. Since the dental pulp is a common colonization and invasion site for *C. albicans*, it is urgent to understand the interaction between *C. albicans* and dental pulp cells. DPSCs are one kind of emerging stem cells used in biological tooth repair and regeneration^49^,^50^. Strategies have to be taken to inhibit the invasion of *C. albicans* to DPSCs. Hence, we used an isolated DPSC line for the evaluation of the AuNP effect on *C. albicans* invasion to host cells. MTT assays revealed that ≤5 ppm AuNPs had no obvious toxicity to DPSCs, and 10–20 ppm of AuNPs only led to slight decrease of DPSC metabolic activity (Fig. S3a). The IC_50_ of AuNPs against DPSCs growth was 63.38 ppm. Moreover, microscopy observation showed that high concentrations of AuNPs (20–80 ppm) resulted in the transformation of DPSCs from adherent cells to planktonic cells (Fig. S3b). This implied that AuNPs might also interact with DPSCs, and thus attenuate the interaction between DPSCs and the substrate surface.

**Figure 4:**
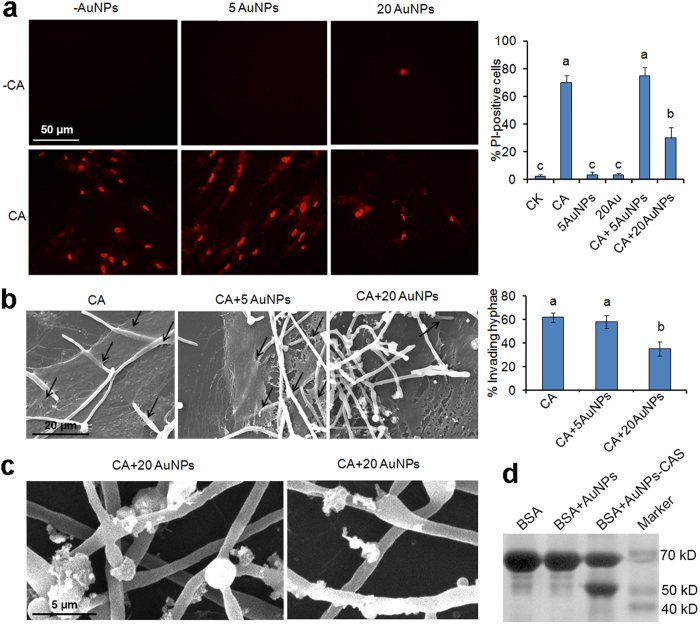
Attenuated *C. albicans* virulence and invasion to DPSCs when co-incubation with AuNPs. (**a**) DPSCs damage after 5 ppm AuNPs (5AuNPs), 20 ppm AuNPs (20AuNPs) alone (−CA) or co-incubated with *C. albicans* cells (CA). The DPSCs after 24 h of treatment were stained with PI and observed by fluorescence microscopy. Columns on the right indicate the statistical results of the percentage of PI-positive DPSCs. (**b**) SEM observation of fungal cells invading into DPSCs when co-incubated with 5 ppm AuNPs (CA + 5AuNPs) or 20 ppm AuNPs (CA + 20AuNPs) or wihout AuNPs (CA). Columns on the right indicate the statistical results of the percentage of invading hyphal cells. (**c**) SEM observation of fungal cells adhered by AuNPs. The fungal cells were co-incubated with DPSCs and 20 ppm AuNPs for 24 h and then observed. The error bars indicate one standard deviations (n = 3). Identical letters indicate no statistical differences among treatments (P < 0.05). (**d**) BSA degradation by AuNPs after adsorption of *C. albicans* culture suspensions. The fungal cells were cultured in BSA medium for induction of protease secretion, then the supernatant containing secreted proteases was obtained. AuNPs were incubated with the supernatants for 24 h, obtaining AuNPs adsorbing secreted proteases (AuNPs-CAS). AuNPs and AuNPs-CAS were added into fresh BSA medium and incubated for 12 h. BSA and its degradation products in the supernatants were then detected by SDS-PAGE and Coomassie Blue staining.

Since the synthesized AuNPs exhibited strong inhibitory effect on the interaction between fungal cells and substrate surface, we hypothesized that they may also affect the interaction between the fungal cells and host cells. PI staining was firstly performed to determine this possible effect. Consistent with the result of MTT assays, AuNP treatment alone with the concentration of 5 ppm or 20 ppm did not cause obvious DPSC death, with only few PI-positive cells observed (Fig. 4a). In contrast, after 6 h of co-incubation, treatment of *C. albicans* cells alone led to severe DPSCs death (>70% of cells died) (Fig. 4a), indicating strong virulence of this pathogen to DPSCs. When combined with *C. albicans* and AuNPs, although 5 ppm of AuNPs had no impact on the death of DPSCs caused by fungal cells, 20 ppm of AuNPs strongly inhibited the death. The death percentage decreased from >70–<30% (Fig. 4a). Therefore, AuNPs at this concentration severely attenuate the virulence of *C. albicans*.

The virulence of *C. albicans* to host cells is commonly attributed to fungal invasion and consequent host cell damage^51^. We then investigated the effect of AuNPs on fungal invasion to DPSCs by scanning electron microscopy (SEM) observation. In the AuNPs-free treatment, abundant hyphal cells (>60%) invaded into DPSCs (indicated by black arrows in Fig. 4b), and treatment with 5 ppm of AuNPs did not affect the invasion. In contrast, 20 ppm of AuNPs significantly inhibited the invasion, and the percentage of invading fungal cells decreased from >60% to about 30% (Fig. 4b). Moreover, detailed SEM observation showed that abundant AuNPs adhered to the hyphal cells in the co-incubation system (Fig. 4c), further confirming the strong interaction between AuNPs and fungal cells. This interaction most likely contributed to the inhibitory effect of AuNPs on fungal invasion.

Secretion of degradative proteases is also required for damage and invasion of *C. albicans* to host cells. Therefore, we also determined the adsorption ability of AuNPs to the secreted proteases using BSA degradation assays. SDS-PAGE staining showed that BSA remained intact when treated by AuNPs alone. However, after incubation with the *C. albicans* culture supernatants (CAS) containing secreted proteases, AuNPs led to remarkable degradation of BSA, with a degradation band produced (Fig. 4d). This indicated that AuNPs had strong adsorption ability to *C. albicans* degradative proteases, which may be also associated with inhibition of AuNPs to invasion.

### AuNPs affect the expression of immune response-related genes in DPSCs

**Figure 5:**
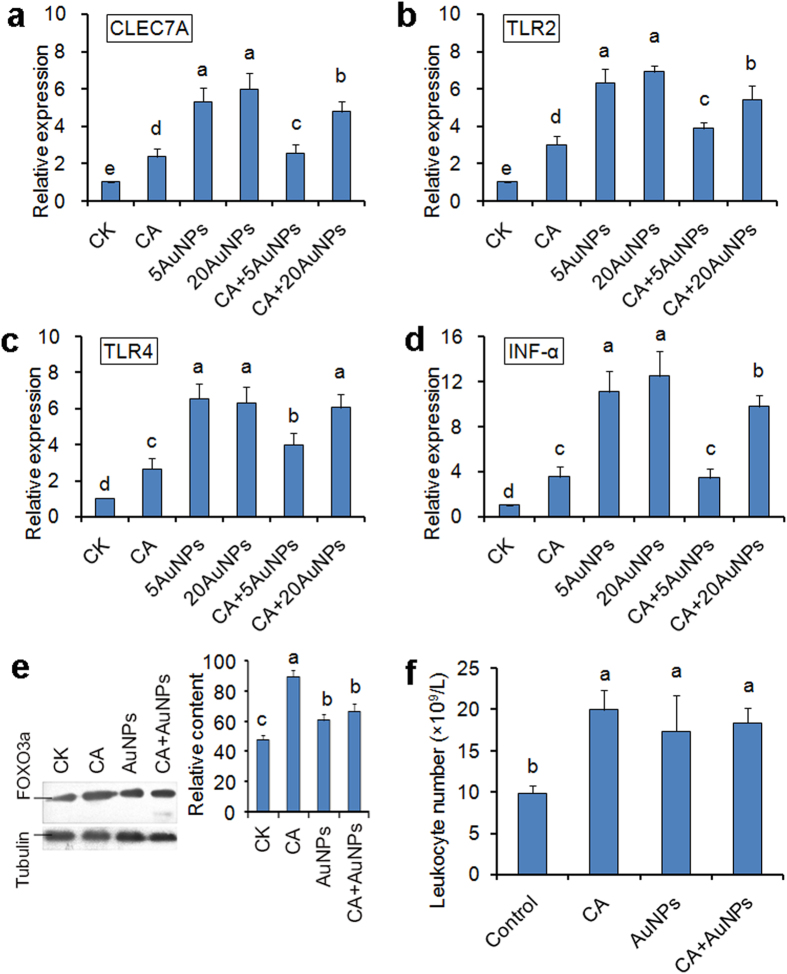
Activation of immune response in DPSCs and mice by *C. albicans* and AuNPs. (**a–d**) Expression levels of immune response-related genes in DPSCs under treatment of *C. albicans* (CA), 5 ppm AuNPs (5AuNPs), 20 ppm AuNPs (20AuNPs), *C. albicans* in combination with 5 ppm AuNPs (CA + 5AuNPs) or *C. albicans* in combination with 20 ppm AuNPs (CA + 20AuNPs). (**e**) Protein levels of FOXO3a in DPSCs. FOXO3a was detected by anti- FOXO3a mono-antibody, and tubulin was used as the normalized protein. Columns on the right indicate the statistical results of FOXO3a contents. (**f**) Leukocyte numbers in the blood of mice after treated by *C. albicans*, AuNPs alone or in combination. 1 mL of *C. albicans* cell suspension (1 × 10^6 ^cells/mL), 1 mL of AuNP solution (5 ppm) or 1 mL of the mixture containing *C. albicans* cells (1 × 10^6 ^cells/mL) and AuNP (5 ppm) was intraperitoneally administered to healthy mice. The blood of the mice was then sampled to determine the number of leukocytes. The error bars indicate one standard deviations (n = 3). Identical letters indicate no statistical differences among treatments (P < 0.05).

The immune system is the most important defense barrier against pathogens^52^. The host cells that are frequently exposed to microbes, such as epithelial cells, dental pulp cells and macrophages, possess complex pattern recognition receptors (PRRs) to perceive microbial touches^53^. Especially, during the interaction with *C. albicans*, several PRRs in those cells which mainly include CLEC7A, TLR2 and TLR4, function as fungus-associated receptors and lead to pro-inflammatory responses, such as the production of INF-α and other immune factors^54^. We then determined the expression of those immune-related genes in AuNPs- and *C. albicans-* treated cells. Real time-PCR (RT-PCR) analysis revealed that *C. albicans* treatment alone up-regulated the expression of all those genes, implying an activation role of this pathogen on immune response. Interestingly, AuNPs treatment resulted in higher expression levels of those genes compared to *C. albicans* treatment (Fig. 5a–d). Moreover, under combined treatment of AuNPs and fungal cells, the expression levels of those genes were also higher than the levels under the treatment of fungal cells alone, but lower than the levels under treatment of AuNPs alone (Fig. 5a–d). We suggested that the fungal cells interacted with AuNPs and attenuated the effect of AuNPs on activating gene expressions. Nevertheless, AuNPs, similar to the clinical pathogen *C. albicans*, strongly activated the expression of immune response-related genes in DPSCs.

We also determined the protein levels of FOXO3a, one of the key transcription factors governing immune response and cell survival^55^. Western blotting revealed that *C. albicans* treatment, AuNP treatment and treatment of *C. albicans* and AuNPs in combination all resulted in an increase of FOXO3a levels (Fig. 5e). This implied that AuNPs and the fungal cells up-regulated the expression of immune response-related genes by regulating FOXO3a.

The *in vivo* effect of AuNPs on immune response was also investigated by leukocyte assays. After intraperitoneally administered by AuNPs alone or in combination with *C. albicans* cells for 5 days, the mice had a significant increase in blood leukocytes, similar to those treated by *C. albicans* cells alone (Fig. 5f). Hence, AuNPs also stimulated immune response in the host.

### Electrostatic attraction is involved in the interaction between AuNPs and targeted cells

**Figure 6:**
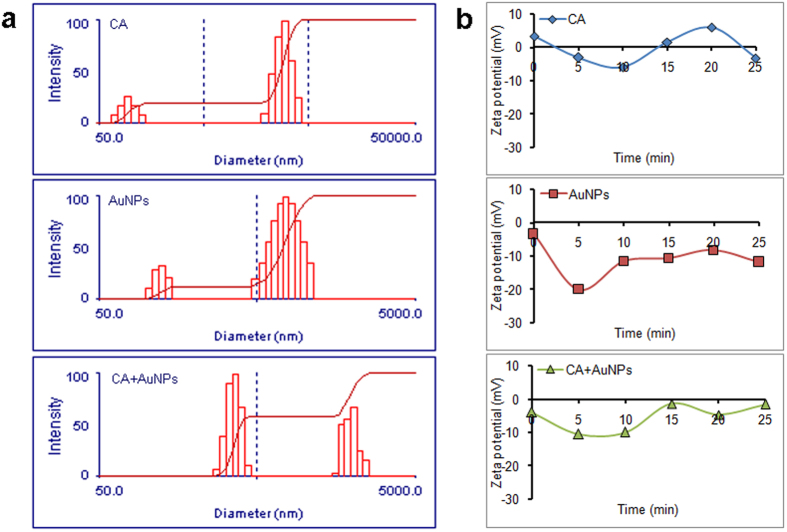
Size distribution and Zeta potential of *C. albicans* alone, AuNPs alone or in combination. (**a**) Size distribution of *C. albicans* alone (CA), AuNPs alone (AuNPs) or *C. albicans* in combination with AuNPs (CA + AuNPs) in the liquid 1640 medium. (**b**) Zeta potential of the tested systems in (**a**).

As implied by inhibition tests, SEM and immune response, the synthesized AuNPs may have strong interaction with the fungal cells. To confirm this interaction, the particle sizes of AuNPs and fungal cells before and after co-incubation were determined by dynamic light scattering (DLS) analyses. In RPMI-1640 medium, *C. albicans* cells showed a major particle size distribution peak at 2,276 nm and a second peak at 95 nm, which represented the whole-cell particles and cell fragments, respectively (Fig. 6a). In contrast, the bimodal distribution of AuNPs displayed a main peak at 679 nm and a second peak at 114 nm. Therefore, AuNPs tended to form aggregates in the medium (Fig. 6a). When fungal cells and AuNPs were mixed, however, the size distribution exhibited two major peaks, one at 363 nm and the other at 2,017 nm (Fig. 6a). The disappearance of the peak at 114 nm for AuNPs and the peak at 95 nm for fungal cell fragments, and the formation of new peaks of enlarged sizes indicated the aggregations of different components after mixing. These results confirmed the existence of interaction between AuNPs and fungal cells, which resulted in further aggregation of them.

Although the interaction between AuNPs and *C. albicans* has been confirmed, it remains unknown how AuNPs interact with *C. albicans* cells. One force that most possibly mediates the interaction between those two particles is electrostatic force. FTIR analysis revealed that there were carbonyl groups on the surface of AuNPs, suggesting that the AuNPs may be negative-potential. On the other hand, *C. albicans* cell surface enriches abundant cell wall proteins, such as GPI-dependent proteins and Pir proteins^56^. These proteins contain both positively charged groups and negatively charged groups. Therefore, we determined the Zeta potential in these systems. Expectedly, the fungal cells alone showed both positive and negative potential. In contrast, AuNPs exhibited completely negative Zeta potential (−9.39 mV). After co-incubation, the system had elevated Zeta potential (−3.45 mV) compared to the single AuNP system (Fig. 6b). These results suggested that electrostatic attraction between AuNPs and fungal cells mediated their interaction, which led to the elevation of Zeta potential.

**Figure 7:**
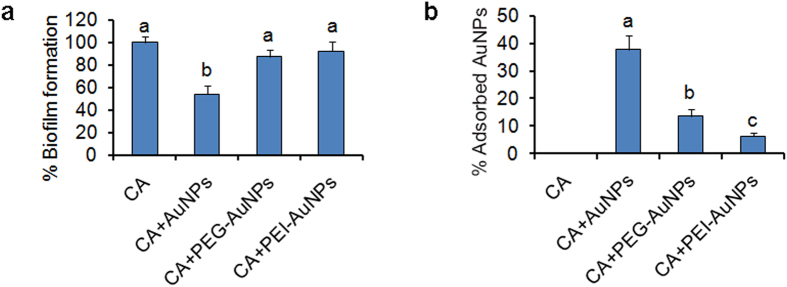
Effect of PEG/PEI coating on inhibition of AuNPs to biolfilm formation and *C. albicans*-AuNPs adsorption. (**a**) *C. albicans* biofilms were formed in RPMI 1640 medium (CA) or the medium containing 5 ppm of AuNPs (CA + AuNPs), PEG-AuNPs (CA + PEG-AuNPs) or PEG-AuNPs (CA + PEI-AuNPs) for 24 h, and the formed biofilms were assessed by the XTT reduction method. (**b**) The formed biofilms were scraped from the microplate bottom and suspended in the previous culture medium. The suspensions were then filtered, and the filtrates were used to determine Au contents by ICP. The error bars indicate one standard deviations (n = 3). Identical letters indicate no statistical differences among treatments (P < 0.05).

To verify that the interaction between AuNPs and *C. albicans* cells is associated with electrostatic attraction, we coated the synthesized AuNPs with the neutral polymer PEG and the cationic polymer PEI, and found that both coats could eliminate the surface negative potential of AuNPs (Fig. S4). Biofilm formation assays and Au adsorption tests were further performed to investigate the effect of coating. Expectedly, compared to the uncoated AuNPs, both PEG-coated and PEI-coated AuNPs had no obvious inhibitory effect on biofilm formation (Fig. 7a). Moreover, PEG- or PEI- coating led to remarkable decrease of adsorbed AuNPs on *C. albicans* cells (Fig. 7b). These results indicated an essential role of the surface negative potential in the interaction between AuNPs and *C. albicans* cells.

## Discussion

Owing to the emergence of drug-resistant microorganisms, it has been an urgency to develop novel antibiotics to improve the efficacy of anti-infection strategies. The application of antimicrobial NMs in inhibiting clinical pathogens is an interesting and exciting field, given their distinctive advantages, including lower acute toxicity, reduced adverse effects and the ability to overcome resistance of pathogens^17^. Although abundant NMs showed strong activity against prokaryotic microorganisms, their activity against eukaryotic microbes remains to be further investigated. In this study, we found that the green-synthesized AuNPs strongly attenuate pathogenic biofilm formation and invasion to host cells, and this activity is associated with the electrostatic interaction between nanoparticles and fungal cells. These findings provide novel insight into anti-microbial NMs during anti-pathogen treatments in the clinic. NMs can be used not only in inhibiting pathogenic bacteria, but also in fighting against fungal pathogens; they can inhibit microorganisms not only by controlling cell growth, but also by impairing biofilm formation and invasion. Especially, since the AuNPs at the concentration of 20 ppm had little toxicity to host cells, but showed strong inhibitory effect on pathogenic biofilm formation and invasion to host cells, these NMs, when prepared with ointment or spray, have a potential application in fighting against oral pathogenic biofilms or damage to host cells.

The anti-microbial activity of AuNPs has been demonstrated in many microbes, including Gram-positive bacteria, Gram-negative bacteria and several fungi. This activity is attributed to special properties of AuNPs in irradiation focusing, strong cationic attractions to the negatively charged plasma membrane of microbes, or conjugation with antimicrobial agents and antibodies. Those properties lead to cell membrane disruption, ROS accumulation and consequent cell death^17^. Herein, we found that the green-synthesized AuNPs at the concentrations of 5–20 ppm showed strong inhibitory effect on pathogenic biofilm formation and invasion. However, no remarkable growth inhibition was observed after AuNP treatments at the concentrations of ≤20 ppm. This suggests that the inhibitory effect is not caused by those properties mentioned above. In contrast, anionic attractions, rather than cationic attractions, of the AuNPs to the cell wall surface of microbes are involved in this effect. Hence, this study uncovers a novel mechanism by which AuNPs fight against pathogenic microbes.

Recently, inhibition of pathogenic biofilm formation has received great attentions. Since biofilm formation depends on cell-to-cell signaling mediated by quorum sensing (QS) molecules, most of the strategies that are devised to inhibit biofilm formation target to and interfere this signaling system. Several QS-related molecules such as farnesol, (S)-4,5-Dihydroxypentane-2,3-dione [(S)-DPD], 4-fluoro-DPD, and carvacrol, have been proved to have inhibitory functions to the QS process in various pathogens^57^,^58^. In this study, however, the synthesized AuNPs which showed strong activity against pathogenic biofilm formation, are obviously not QS-related molecules. One possible mechanism is that the strong electrostatic attractions between AuNPs and cell wall surface of the pathogens interrupt adhesin-mediated interaction between the pathogenic cells and the substrate surfaces, which is confirmed by Hwp1-GFP observations in this study.

Similarly, inhibition of the AuNPs to invasion may also be attributed to the electrostatic interactions. For *C. albicans* and many other pathogens, the secretion of extracellular hydrolytic enzymes such as secreted aspartyl proteinases (Sap), phospholipases, and lipases, plays important roles in damaging and invading host cells^59^,^60^. This suggests that the synthesized AuNPs may bind to hydrolytic enzymes by electrostatic forces, leading to inactivation of those enzymes which are essential for invasion. Consequently, AuNPs prevent pathogens from invading into host cells, and keep the host cells from pathogenic damage.

Commonly, the host immune system functions in protecting the body from invasion of pathogens, which is stimulated when the pathogens interact with the host cells, leading to activation of immune response pathways and up-regulation of related genes. Previous studies have shown that peptide-conjugated AuNPs were identified by the macrophages and subsequently activate immune response, while peptide or AuNPs alone were not identified^61^. Interestingly, the synthesized AuNPs in this study not only inhibit pathogens, but also activate the expression of immune response-related genes in the host cells. This indicates that these particles, similar to the pathogens, can be recognized by immune system and activate immune response. This may be attributed to the surface conjugation of AuNPs with the components in the apple extract, leading to strong interaction between AuNPs and immune receptors of the host cells and subsequent activation of immune response pathways.

Toxicity of AuNPs to normal mammalian cells is important issue. Evidence have indicated that AuNPs caused toxicity to the mammalian cells after internalization, which is associated with ROS accumulation, DNA fragmentation and mitochondrial damage^62^,^63^. Consistently, in this study, we also found that the synthesized AuNPs showed an obvious toxicity to human DPSCs when the concentration reached up to 40 ppm. In contrast, the AuNPs had no significant inhibitory effect on *C. albicans* growth even the concentration reached to 160 ppm, although AuNPs at this concentration strongly attenuated biofilm formation. The difference in response to AuNPs between the fungal cells and human cells might be attributed to the distinct cell structures between these two kinds of cells. Especially, the fungal cells have the cell wall, which prevents the internalization of extracellular nanoparticles into the cells, limiting the interaction between the nanoparticles and intracellular organelles and biomolecules^64^. Hence, the nanoparticles had lower toxicity to fungal cells than to mammalian cells.

In conclusion, this study revealed the strong inhibitory effect of as-synthesized AuNPs on pathogenic biofilm formation and invasion to host cells. Moreover, this effect does not result from growth inhibition or cell death, but is mediated by the strong electrostatic interaction between nanoparticles and pathogenic cells. These findings shed novel light on the application of nanomaterials in fighting against clinical pathogens.

## Methods

### Synthesis and characterization of AuNPs

AuNPs were synthesized by reducing HAuCl_4_ to Au^0^ with two methods. One is based on apple extract according to Sharma’s method with modification (named AuNP-S)^65^. Briefly, 2 ml of HAuCl_4_ solution (10 mM, prepared in dH_2_O) were mixed with 50 ml of apple extract (pre-filtered by four-layer filters), and then the mixture was shaken with 160 rpm at 80 °C for 12 h. The AuNPs were then harvested by centrifugation at 12000 rpm for 10 min, washed with alcohol for several times, and dried at room temperature. The other is chemical reduction of HAuCl_4_ by dissolved trisodium citrate with Polte’s method (named AuNPs-P)^66^.

General morphology of the synthesized AuNPs was characterized by transmission electron microscopy (TEM, Tecnai G^2^ F-20, FEI, USA). The crystal structure and composition of the samples were characterized by X-ray diffraction (XRD, D/max-2500, Japan). The absorption property was assayed by UV/Vis spectrometry (Enspire, Perkinelmer, USA). The surface groups were detected by FTIR (Bio-Rad, FTS6000, USA).

### Preparation of AuNP solutions

The solutions of synthesized AuNPs were prepared in fresh RPMI-1640 medium (RPMI-1640 powder (Sigma, USA) 1%, 3-(N-morpholino) propanesulfonic acid (Sigma, USA) 0.418%, uridine 80 ppm, pH 7.4) with the initial concentration of 10 000 ppm. The stock solution was then sonicated for 30 min (AS3120, Autoscience, China) and 2-fold diluted using the same medium, obtaining the following concentrations of AuNPs, 5, 10, 20, 40, 80, 160 and 320 ppm.

### PEG/PEI coating of AuNPs

To prepare PEG- or PEI-coated AuNPs, the synthesized AuNPs-S were dissolved in distilled water to a concentration of 5 000 ppm. polyethylene glycol (PEG, Mw = 3350) or polyethyleneimine (PEI, Mw = 10,000) was then added into the solutions to the final concentrations of 1.0 mg/ml. The mixtures were incubated at 30 °C with gently shaking for 24 h, and then were centrifuged at 12,000 rpm for 10 min. The pellets were washed with dH_2_O for three times and stored in 1 mM NaCl for further experiments.

### Strains, cell lines and growth conditions

The *C. albicans* strains used in this study are listed in Table S1. The strains SC5314, BWP17 and clinical isolated strains were used for both growth inhibition test and biofilm formation assay. To observe the distribution of the fungal adhesin Hwp1, the *HWP1* gene was internally fused with GFP fragment (amplified form the plasmid pGFP-NAT1), cloned into the plasmid pAU34M, obtaining the plasmid pAU34M-Hwp1-GFP. The plasmid was then digested with *Bgl* II and transformed into BWP17, obtaining the Hwp1-localization strain NKFR1. Normally, the fungal cells were cultured in liquid YPD medium (yeast extract 1%, peptone 2%, glucose 2%) at 30 °C with shaking. During hyphal induction and biofilm formation, the fungal cells were cultured in liquid RPMI-1640 medium at 37 °C. The clinical *C. albicans* strains, CL001, CL016, CL018, CL105 and CL201, and *P. aeruginosa* strain CL211 were isolated in Tianjin Third Central Hospital, respectively. All clinical strains were identified by internal transcribed spacer (ITS) sequencing (for *C. albicans* strains) or 16S rDNA sequencing (for *P. aeruginosa* strain).

The dental pulp stem cells (DPSCs) were originally isolated from the dental pulp of a lost tooth from a healthy child, and suspended in RPMI-1640 medium +10% fetal bovine serum (FBS). The cell suspensions were then added into 24-well polystyene microplates and cultured in a humidified incubator at 37 °C in 5% CO_2_ for 2 days.

### Growth inhibition tests

Growth inhibition tests of AuNPs against *C. albicans* were performed according to the CLSI standard except that the fungal cells were cultured at 37 °C. The tested AuNPs concentrations were 0, 5,10, 20, 40, 80 and 160 ppm, respectively. To test the inhibitory effect of AuNPs to DPSCs, DPSCs were suspended in RPMI-1640 medium +5% FBS containing AuNPs with the concentrations as described above. The mixture was then cultured at 37 °C in 5% CO_2_ for 2 days, and cellular metabolic activity was determined by MTT assays.

### Hyphal induction

Liquid RPMI-1640 medium was used to induce *C. albicans* hyphae. This medium contains several molecules that can activate the signaling pathways leading to expression of hypha-specific genes and consequent hyphal development, such as HCO3^−^ and methionene. Overnight cultured *C. albicans* yeast-type cells were washed with PBS and suspended with the OD_600_ of 0.1 in RPMI-1640 medium containing AuNPs with the indicated concentrations. The suspensions were then incubated with shaking at 37 °C for 24 h. Cells were harvested and observed by a light microscope (Leica, Germany).

### Biofilm formation

*C. albicans* biofilms formed in polystyrene microtiter plates (Corning Inc., USA). Briefly, overnight cultured fungal cells were suspended with the OD_600_ of 0.1 in RPMI-1640 medium containing AuNPs at different concentrations. 100 μL of the suspensions were then added into 96-well microtiter plates. The plates were covered with lids and incubated at 37 °C. After 24 h of incubation, the plates were washed with PBS, and the biofilm activity was detected by XTT (2,3-bis (2-methoxy-4-nitro-5-sulfophenyl)-2H-tetrazolium-5- carboxanilide) reduction assays. Biofilms were also observed by SEM. The biofilm-contained wells were washed with PBS buffer, fixed overnight with glutaraldehyde, dehydrated with ethanol, and dried in vacuum desiccators. The obtained samples were coated with gold and observed by a scanning electron microscope (QUANTA 200, Czech). *P. aeruginosa* biofilms were formed with similar methods, except that LB medium (yeast extract 0.5%, NaCl 1%, tryptone 1%) was used.

### Confocal microscopy

The Hwp1-GFP fusion protein was visualized by confocal microscopy using the layer-scanning model. AuNPs-treated or -untreated *C. albicans* biofilms were washed with PBS, fixed with 4% formaldehyde, and observed with a confocal microscope (LSM710, Zeiss, Germany) using the GFP filter set. The total scanning distance was set with 8 μm, and the distance between two adjacent layers was 2 μm.

### Inhibition tests of *C. albicans* invasion to DPSCs

To determine the inhibition effect of AuNPs on *C. albicans* invasion, *C. albicans* cells were overnight cultured in YPD medium, and suspended in RPMI-1640 +10% FBS medium containing AuNPs at different concentrations. DPSCs were cultured in 24-well microplates for 2 days as described above, and the cell supernatant was removed. 1 mL of fungal suspensions was then added into each well. The microplates were cultured at 37 °C in 5% CO_2 _for further 6 h. The damaged DPSCs were counted after PI staining (final PI concentration 2 μg/mL). The cells in the wells were fixed and observed by SEM as described in biofilm formation. The total hyphal cells and the invading hyphal cells were counted, and the percentage of invading hyphae was calculated with the number of invading hyphae divided by that of total hyphae. At least 20 fields were determined.

### BSA (bovine serum albumin) degradation tests

To determine the adsoption ability of AuNPs to *C. albicans* secreted proteases, the fungal cells were cultured in BSA medium (glucose 1%, BSA 1%, trace element solution 0.1%, vitamin solution 0.1%, histidine 100 μg/ml, arginine 100 μg/ml, uridine 100 μg/ml) at 30 °C with shaking for induction of protease secretion. The cultures were centrifuged at 12,000 rpm for 10 min, obtaining the supernatant containing secreted proteases. AuNPs were then added into the supernatant to a final concentration of 1,000 ppm, incubated at 30 °C with shaking for 24 h. The AuNPs were harvested with centrifugation at 12,000 rpm for 10 min, washed three times with BSA medium, obtaining AuNPs adsorbing secreted proteases (AuNPs-CAS). AuNPs and AuNPs-CAS were added into fresh BSA medium to a final concentration of 20 ppm, respectively. The mixtures were incubated at 30 °C with shaking for 12 h, and centrifuged at 12,000 rpm for 10 min. 20 μl of the supernatants were used for SDS-PAGE. BSA and its degradation products were detected in the PAGE with Coomassie Blue staining.

### Real time-PCR

For *C. albicans* cells, RT-PCR was performed to detect expression of hypha-related genes under hyphal development and biofilm formation. *C. albicans* hyphal development and biofilm formation proceeded as described above. The fungal cells were then harvested and suspended in the Trizol agent (Dingguo, China), and were lysed with glass beads by vortexing. Total RNAs were extracted from the lysates by isopropanol precipitation, and were used for reverse transcriptional synthesis of cDNA by Oligo (dT)-primed RT reagent Kit (Promega, MADISON, USA). The TransStart Green qPCR Supermix Kit (TRANSGEN, Beijing) were used for RT-PCR. Transcription levels of the hypha-associated genes, *HWP1, ALS3* and *ECE1*, were normalized against the levels of *ACT1* in different samples. These genes were amplified by the Eppendorf Real-Time PCR Detection System (Realplex2, Eppendorf, USA) with the following forward and reverse primers ACT1-5RT, ACT1-3RT, HWP1-5RT, HWP1-3RT, ALS3-5RT, ALS3-3RT, ECE1-5RT, ECE1-3RT (Table S2), respectively. The expression levels of *HWP1, ALS3* and *ECE1* were analyzed using the Eppendorf Real-time PCR Software.

For DPSCs, RT-PCR was performed to investigate the expression levels of immune response-related genes. *C. albicans* cells and DPSCs were co-incubated in RPMI-1640 +10% FBS medium containing AuNPs as described above. The supernatant were removed, and DPSCs were lysed by the Trizol agent. Total RNAs and cDNA were prepared, and transcription levels of the immune response-related genes, CLEC7A, TLR2, TLR4 and INF-α, were normalized against the levels of GAPDH in different samples. These genes were detected with the following forward and reverse primers, CLEC7A-5RT, CLEC7A-3RT, TLR2-5RT, TLR2-3RT, TLR4-5RT, TLR4-3RT, INF-α-5RT, INF-α-3RT (Table S2), respectively. All samples were taken in triplicate independent experiments.

### Western blotting

To detect the protein levels of FOXO3a, *C. albicans* cells and DPSCs were co-incubated in RPMI-1640 +10% FBS medium containing AuNPs as described above. DPSCs were then lysed by RIPA buffer containing protease inhibitor cocktail (Roche, Switzerland). The total proteins were prepared by vortex and centrifugation. FOXO3a in the samples were detected by Western blotting using the FOXO3a mono-antibody (Sangene, China). α-Tubulin was also detected using the tubulin mono-antibody (Abcam, USA) as a control.

### Interaction between *C. albicans* and AuNPs

Overnight cultured *C. albicans* were suspended in RPMI-1640 medium with the initial OD_600_ of 0.1. AuNPs with the concentration of 5 ppm were prepared in the same medium. Equal volumes of fungal cell suspension and AuNPs solution were mixed, obtaining the *C. albicans-*AuNPs mixture. Size distribution and Zeta potential of the fungal cell suspension, AuNPs solution and the *C. albicans-*AuNPs mixture were then determined using ZetaPALS (ZETAPALS/BI-200SM, Brookhaven, USA).

### Mouse leukocyte assays under treatment of *C. albicans* and AuNPs

To *in vivo* assess the effect of *C. albicans* and AuNPs on mouse immune response, 4–6 week-old healthy Kunming female mice were used. 1 mL of *C. albicans* cell suspension (1 × 10^6 ^cells/mL, fixed by 4% formaldehyde), 1 mL of AuNP solution (5 ppm) or 1 mL of the mixture containing *C. albicans* cells (1 × 10^6 ^cells/mL, fixed by 4% formaldehyde) and AuNP (5 ppm) was intraperitoneally administered to healthy mice. The blood of the mice was then sampled from the tail vein, and the number of leukocytes in the blood was measured using automatic blood analyzer (MC 6200, ICUBIO, China).

### Biofilm-AuNP adsorption assays

To determine the contents of AuNPs adsorbed by *C. albicans* biofilms, *C. albicans* biofilms were formed in RPMI 1640 medium (CA) or the medium containing 5 ppm of AuNPs (CA + AuNPs), PEG-AuNPs (CA + PEG-AuNPs) or PEG-AuNPs (CA+PEI-AuNPs) for 24 h. The formed biofilms were then scraped from the microplate bottom and suspended in the previous culture medium. The suspensions were vortexed for 2 min to release the un-adsorbed AuNPs from the biofilms, and filtered using microfiltration membrane (nominal pore size = 0.4 μm), obtaining the filtrates. The filtrates were digested with 30% HNO_3_, and Au contents in the digestion liquid were determined with inductively coupled plasma−mass spectrometry (ICP−MS, PerkinElmer, ELAN DRC-e). The percent of adhered Au was calculated by (the concentration of AuNPs in the initial culture medium − that in the filtrates)  ×  100/the concentration of AuNPs in the initial culture medium).

### Statistical analysis

The experiments were performed in triplicate, and the values represent the means + standard deviations (SD). Significant differences between the treatments were determined using one-way ANOVA (*P* < 0.05). Statistical analysis and IC_50_ calculation were performed by the SPSS software (Version 20, IBM, USA).

### Ethics statement

All mouse experiments were approved by the Institutional Animal Care and Use Committee of Nankai University. These experiments were performed in accordance with the relevant guidelines of this committee. All mice had free access to food and water throughout the experiments. The mice were humanely sacrificed via carbon dioxide euthanasia, and all efforts were made to minimize their suffering.

## Additional Information

**How to cite this article**: Yu, Q. *et al*. Inhibition of gold nanoparticles (AuNPs) on pathogenic biofilm formation and invasion to host cells. *Sci. Rep.* **6**, 26667; doi: 10.1038/srep26667 (2016).

## Supplementary Material

Supplementary Information

## Figures and Tables

**Table 1:** Inhibition effect of AuNPs on *C. albicans* growth and biofilm formation.

**Strains**	**Species**	**AuNPs-S IC**_**50**_ **against growth (ppm)**	**AuNPs-P IC**_**50**_ **against growth (ppm)**	**AuNPs-S IC**_**50**_ **against biofilm formation (ppm)**	**AuNPs-P IC**_**50**_ **against biofilm formation (ppm)**
SC5314	*C. albicans*	>160	>160	6.316 ± 1.430	7.422 ± 1.098
BWP17	*C. albicans*	>160	>160	5.148 ± 1.697	6.034 ± 2.151
NKFR1	*C. albicans*	>160	>160	5.344 ± 1.284	5.980 ± 1.452
CL001	*C. albicans*	>160	>160	9.240 ± 1.723	9.342 ± 1.870
CL016	*C. albicans*	>160	>160	6.489 ± 0.895	6.974 ± 1.217
CL018	*C. albicans*	>160	>160	8.862 ± 1.748	8.753 ± 1.032
CL105	*C. albicans*	>160	>160	10.004 ± 1.625	10.354 ± 1.542
CL201	*C. albicans*	>160	>160	8.294 ± 1.398	9.532 ± 1.659
CL211	*P. aeruginosa*	68.56 ± 5.941	75.01 ± 7.905	6.937 ± 2.310	6.851 ± 1.504
