# Supplementary material for: Inhibition of gold nanoparticles (AuNPs) on pathogenic biofilm formation and invasion to host cells
Source: Sci Rep. 2016 May 25;6:26667. doi: 10.1038/srep26667 (PMC4879543; doi:10.1038/srep26667)
Supplement: Supplementary Information [file srep26667-s1.doc]

**Additional information**

**Inhibition of gold nanoparticles (AuNPs) on pathogenic biofilm formation and invasion to host cells**

Qilin Yu ^1^, Jianrong Li ^1^, Yueqi Zhang ^1^, Yufan Wang ^2^, Lu Liu^3^, Mingchun Li^1^

^1^Key Laboratory of Molecular Microbiology and Technology, Ministry of Education, Department of Microbiology, Nankai University, Tianjin, PR China. ^2^Clinical Laboratory, Tianjin Third Central Hospital, Tianjin, PR China. ^3^Tianjin Key Laboratory of Environmental Remediation and Pollution Control, College of Environmental Science and Engineering, Nankai University, Tianjin, PR China. Correspondence and requests for materials should be addressed to M.C.L. (email: [nklimingchun@163.com](mailto:nklimingchun@163.com))

**Table S1.** **Strains used in this study**

| Strains | Genotype | Source |
| --- | --- | --- |
| *C. albicans* |  |  |
| SC5314 | wild type | ATCC |
| BWP17 | *ura3Δ::λimm434/ura3Δ::λimm434 his1::hisG/his1::hisG arg4::hisG/arg4::hisG* | Dana Davis (32) |
| NKFR1 | *ura3Δ::λimm434/ura3Δ::λimm434 his1::hisG/his1::hisG arg4::hisG/arg4::hisG HWP1-GFP* | This study |
| CL001 | wild type, clinical isolation | This study |
| CL016 | wild type, clinical isolation | This study |
| CL018 | wild type, clinical isolation | This study |
| CL105 | wild type, clinical isolation | This study |
| CL201 | wild type, clinical isolation | This study |
| *P. aeruginosa* |  |  |
| CL211 | wild type, clinical isolation | This study |

**Table S2.** Primers used in this study

| Primers | Sequence |
| --- | --- |
| ACT1-5RT | GGTAGACCAAGACATCAAGG |
| ACT1-3RT | CCGTGTTCAATTGGGTATCT |
| HWP1-5RT | TGTCTACACTACATTCTGTC |
| HWP1-3RT | AGGAATAGATGGTTGTGAAC |
| ECE1-5RT | CCAAGCACCTACTGTTCC |
| ECE1-3RT | GATACCAGCAACAACAGAAT |
| ALS3-5RT | CTCATTACACCAACCATACA |
| ALS3-5RT | GGATTCTGTGGTTGTAGTAT |
| GAPDH-5RT | GAAGGTGAAGGTCGGAGTC |
| GAPDH-3RT | GAAGATGGTGATGGGATTTC |
| CLEC7A-5RT | GGAAGCAACACATTGGAGAATGG |
| CLEC7A-3RT | CTTTGGTAGGAGTCACACTGTC |
| TLR2-5RT | GCCTCTCCAAGGAAGAATCC |
| TLR2-3RT | TCCTGTTGTTGGACAGGTCA |
| TLR4-5RT | AGTTTCCTGCAATGGATCAAGG |
| TLR4-3RT | CTGCTTATCTGAAGGTGTTGCAC |
| INF-α-5RT | TCCAGGCGGTGCCTATGT |
| INF-α-3RT | CACCCCGAAGTTCAGTAGACAGA |

**
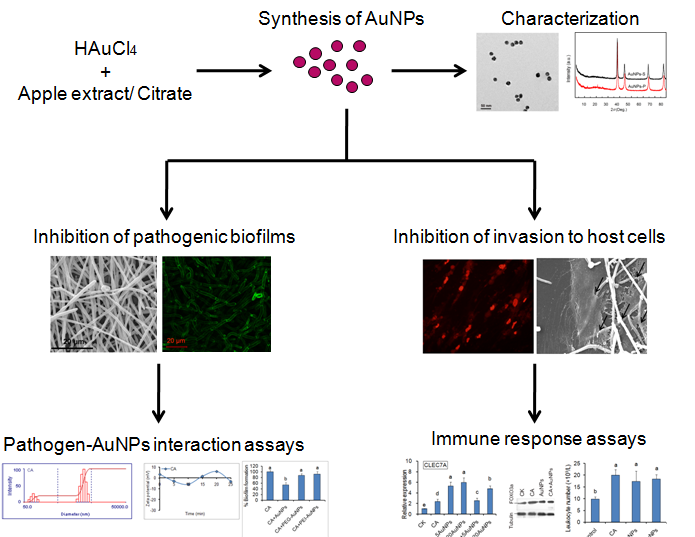
**

**Figure S1.** A schematic of this work.


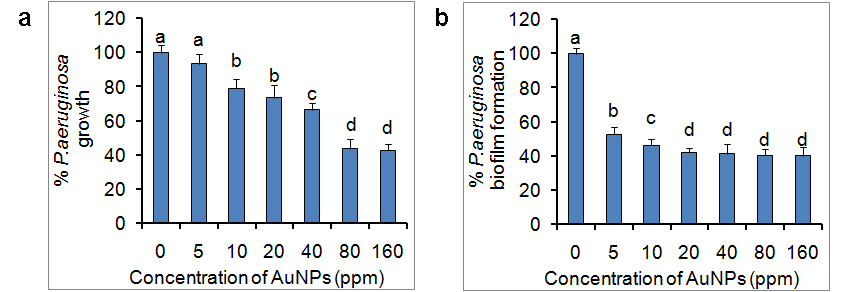


**Figure S2. Inhibition of AuNPs to *P. aeroginosa* growth and biofilm formation.** (a) Metabolic activity of *P. aeruginosa* after treatment of AuNPs with different concentrations. (b) Metabolic activity of formed *P. aeruginosa* biofilms after AuNPs treatment. The error bars indicate one standard deviations (n = 3). Identical letters indicate no statistical differences among treatments (P < 0.05).


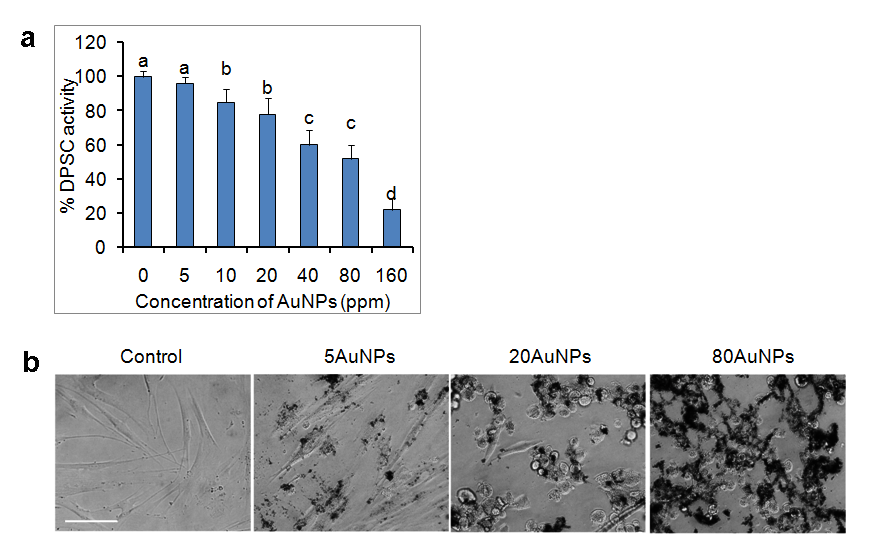


**Figure S3. Growth inhibition of AuNPs to DPSCs.** (a) The activity of DPSCs measured by MTT assays after 24 h of AuNP treatment with different concentrations. (b) Microscopic observation of DPSCs without AuNP treatment (Control) or those after treatment of 5 ppm AuNPs (5AuNPs), 20 ppm AuNPs (20AuNPs) or 80 ppm AuNPs (80AuNPs). Bar = 50 μm. The error bars indicate one standard deviations (n = 3). Identical letters indicate no statistical differences among treatments (P < 0.05).


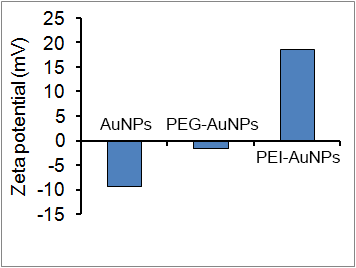


**Figure S4. Zeta potential of the synthesized AuNPs, PEG-coated AuNPs (PEG-AuNPs) and PEI-coated AuNPs (PEI-AuNPs).**
